# Clustering of subpatent infections in households with asymptomatic rapid diagnostic test-positive cases in Bioko Island, Equatorial Guinea independent of travel to regions of higher malaria endemicity: a cross-sectional study

**DOI:** 10.1186/s12936-021-03844-6

**Published:** 2021-07-12

**Authors:** Dianna E. B. Hergott, Jennifer E. Balkus, Guillermo A. García, Kurtis R. Cruz, Annette M. Seilie, Haley Masters, Akum A. Aveika, Marcos Mbuilto Iyanga, Jeremias Nzamio Mba Eyono, Brandon L. Guthrie, Sean C. Murphy

**Affiliations:** 1 Department of Epidemiology, University of Washington School of Public Health, Seattle, WA USA; 2 Department of Global Health, University of Washington School of Public Health, Seattle, WA USA; 3 Medical Care Development International, Silver Spring, MD USA; 4 Department of Laboratory Medicine and Pathology, University of Washington, Seattle, WA USA; 5 Center for Emerging and Re-Emerging Infectious Diseases, University of Washington, Seattle, WA USA; 6 Department of International Health, Johns Hopkins University, Baltimore, MD USA; 7 Medical Care Development International, Malabo, Equatorial Guinea; 8 Department of Microbiology, University of Washington, Seattle, WA USA

**Keywords:** *Plasmodium falciparum*, Malaria, Clustering, Subpatent infection, Importation

## Abstract

**Background:**

Prevalence of falciparum malaria on Bioko Island remains high despite sustained, intensive control. Progress may be hindered by high proportions of subpatent infections that are not detected by rapid diagnostic tests (RDT) but contribute to onward transmission, and by imported infections. Better understanding of the relationship between subpatent infections and RDT-detected infections, and whether this relationship is different from imported *versus* locally acquired infections, is imperative to better understand the sources of infection and mechanisms of transmission to tailor more effective interventions.

**Methods:**

Quantitative reverse transcriptase polymerase chain reaction (qRT-PCR) was performed on a sub-set of samples from the 2015 Malaria Indicator Survey to identify subpatent infections. Households with RDT(+) individuals were matched 1:4 with households with no RDT(+) individuals. The association between living in a household with an RDT(+) individual and having a subpatent infection was evaluated using multivariate hierarchical logistic regression models with inverse probability weights for selection. To evaluate possible modification of the association by potential importation of the RDT(+) case, the analysis was repeated among strata of matched sets based on the reported eight-week travel history of the RDT(+) individual(s).

**Results:**

There were 142 subpatent infections detected in 1,400 individuals (10.0%). The prevalence of subpatent infections was higher in households with *versus* without an RDT(+) individual (15.0 *vs* 9.1%). The adjusted prevalence odds of subpatent infection were 2.59-fold greater (95% CI: 1.31, 5.09) for those in a household with an RDT(+) individual compared to individuals in a household without RDT(+) individuals. When stratifying by travel history of the RDT(+) individual, the association between subpatent infections and RDT(+) infections was stronger in the strata in which the RDT(+) individual(s) had not recently travelled (adjusted prevalence odds ratio (aPOR) 2.95; 95% CI:1.17, 7.41), and attenuated in the strata in which recent travel was reported (aPOR 1.76; 95% CI: 0.54, 5.67).

**Conclusions:**

There is clustering of subpatent infections around RDT(+) individual(s) when both imported and local infection are suspected. Future control strategies that aim to treat whole households in which an RDT(+) individual is found may target a substantial portion of infections that would otherwise not be detected.

**Supplementary Information:**

The online version contains supplementary material available at 10.1186/s12936-021-03844-6.

## Background

Despite 17 years of intensive malaria control efforts funded at more than three times the regional average, the prevalence of falciparum malaria on Bioko Island, Equatorial Guinea, as determined through rapid diagnostic tests (RDTs) in cross-sectional surveys, remains high at 17% [1]. The prevalence is highly heterogenous [2], with some areas reporting prevalence < 5% while others > 25% [3]. Such heterogeneity is perplexing; some of the highest burdens are reported in urban areas where improved housing conditions and fewer mosquito breeding sites typically result in lower malaria prevalence [4–7]. To move Bioko Island towards malaria elimination, it is imperative to better understand the sources of infection and mechanisms of transmission to tailor more effective interventions.

Both clinical and active case detection strategies for malaria diagnosis and treatment typically rely on RDTs and/or microscopy. While these diagnostic approaches are highly sensitive for higher density infections (higher concentration of parasites per unit of blood), they fail to capture lower density infections that are below the limit of detection [8]. Such subpatent infections are highly prevalent in endemic areas [8] and contribute to onward transmission [9]. More sensitive molecular techniques, such as polymerase chain reaction (PCR) and reverse transcription PCR (RT-PCR), are more costly and require laboratory infrastructure not routinely available in endemic areas, making their widespread use for front-line diagnostics improbable. However, if such tools can be used to identify relationships and patterns between subpatent and patent infections, it may enable the development of intervention strategies to target subpatent infections without use of complex testing. For example, a recent meta-analysis with data from 41 malaria endemic countries showed that household clustering of subpatent infections around symptomatic or asymptomatic patent infections exists in areas where local malaria transmission occurs depending on site’s overall endemicity [10].

**Fig. 1:**
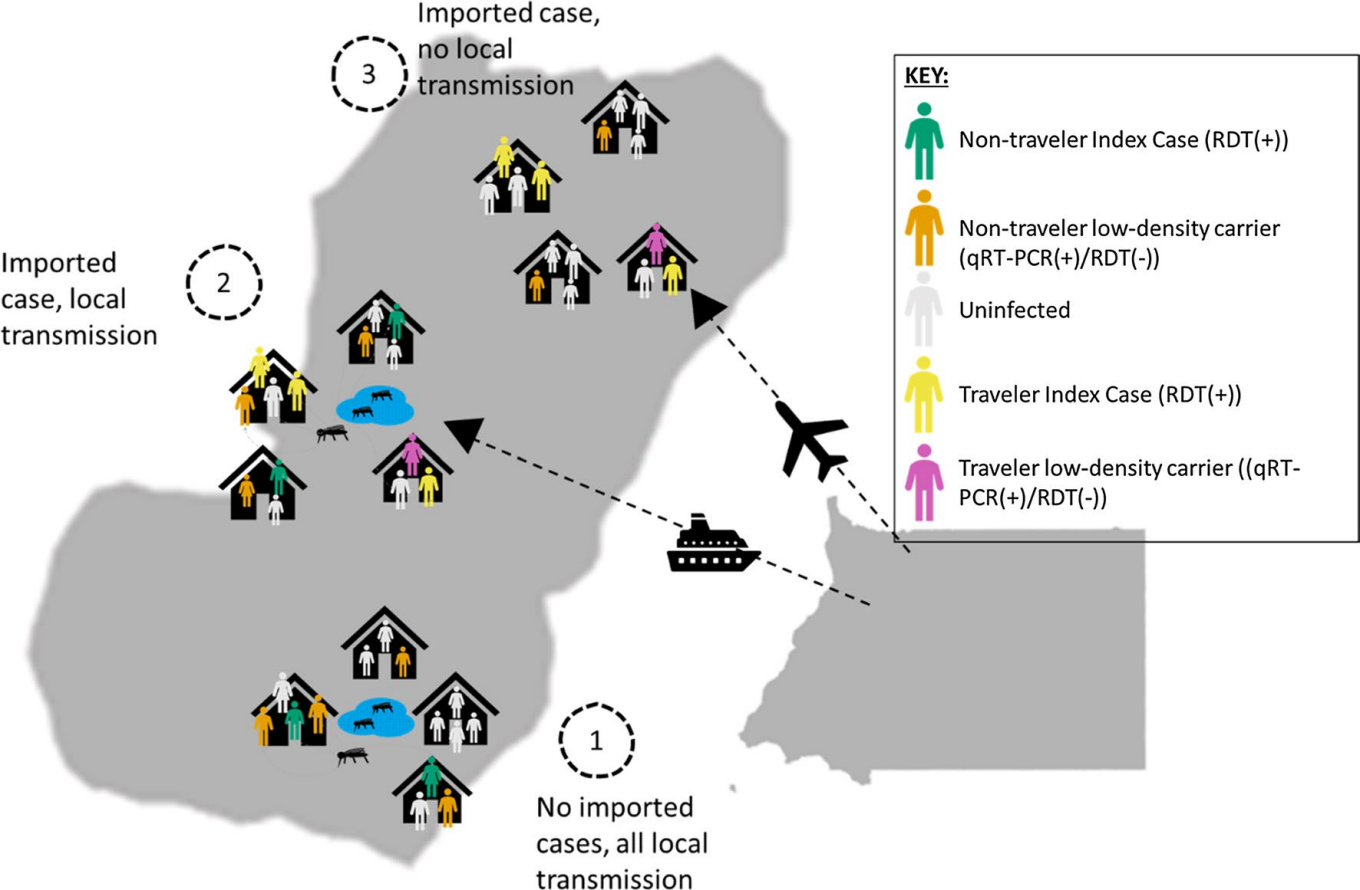
Schematic diagram showing the three possible transmission profiles hypothesized to exist on Bioko Island

The malaria transmission dynamics on Bioko Island are complicated by the high frequency of travel between the island (relatively low prevalence) and the mainland portion of the country (much higher prevalence, > 30%) [11, 12]. Importation of malaria cases from higher burden neighbours threatens elimination programmes in several endemic areas [13–17]. A recent analysis of Bioko Island suggested that the high prevalence in urban areas was mainly attributed to importation of cases, rather than local transmission, and thus control measures targeting travellers were proposed [12, 18]. However, it is unclear if household clustering of subpatent infections exists in areas that have both low receptivity to onward transmission and a high proportion of imported cases. For *Plasmodium* infection to propagate in a community, there must be a certain level of receptivity at that locale [16], which is dependent on the presence of available mosquitoes and human reservoirs. Therefore, network clusters of infections are expected in areas where local transmission is occurring. Local transmission can either be propagated from other locally occurring infections (Route 1 of Fig. 1) or from an imported infection (Route 2 of Fig. 1). Control strategies targeting travellers will be most successful in reducing the overall malaria burden in areas where imported infections contribute substantially to sustained transmission. However, these strategies may be less impactful on reducing island-wide transmission if such imported infections occur in areas where the conditions are not conducive to onward transmission (Route 3 of Fig. 1). The association between subpatent infections, RDT(+) infections, and importation in Bioko Island, is unknown.

Given the potential role of both subpatent infections and travel on malaria transmission dynamics, this study utilized samples and data from the annual Malaria Indicator Survey to evaluate whether subpatent infections are more common in Bioko Island households with an RDT(+) individual than those without an RDT(+) individual, and whether the association was modified by the potential importation of the RDT(+) infection in a traveller. It was hypothesized that the greatest proportion of subpatent infections would be found in households with RDT(+) individuals with no recent travel history.

## Methods

### Study setting and original data collection

Bioko Island, the largest island of Equatorial Guinea, is approximately 2000 sq km and has ~ 335,000 inhabitants as of 2015, the majority of whom (80%) live in the urban district of Malabo in the northern region of the island [19]. Malaria transmission occurs year-round. Since 2004, the National Malaria Control Programme (NMCP) has launched an intensive malaria control strategy in collaboration with the Bioko Island Malaria Control Programme (BIMCP), which includes indoor residual spraying (IRS), distribution of long-lasting insecticidal nets (LLINs), distribution of free anti-malarials for uncomplicated and complicated malaria, intermittent preventive treatment in pregnancy (IPTp), entomological monitoring, a behaviour change communication programme, and enhanced malaria testing strategies [3, 20, 21].

The Malaria Indicator Survey (MIS) is carried out annually on Bioko Island to track malaria prevalence; the present analysis uses data and samples collected during the 2015 MIS. The sampling strategy and study design were previously described [3]. In brief, households were randomly selected by an independent statistician from each of the 284 communities on the island that had at least 20 households registered in the 2015 census [22]. A trained enumerator interviewed the head of household to gather demographic information on all household members, household and individual characteristics, as well as malaria knowledge and household behaviors relating to malaria prevention. All members of the household who were present at the time of the survey and provided consent were invited to be tested for malaria parasitaemia and two dried blood spots (DBS) were collected on filter paper and stored at ambient temperature in desiccant for later analysis. Participants with malaria parasitaemia were provided with artemisinin-combination therapy (ACT) by a Ministry of Health and Social Welfare (MoHSW) nurse per the national malaria treatment policy. Those with haemoglobin < 8 g/dL or who were febrile were referred to a local clinic for appropriate follow-up and treatment.

### Laboratory methods

Malaria parasitaemia in MIS was assessed using a CareStart Malaria HRP2/pLDH RDT (Access Bio, Somerset, NJ, USA). For this analysis, only *Plasmodium falciparum* parasitaemia was considered, indicated by the presence of the HRP2 band, either with or without the pLDH band. Subpatent malaria infections were detected using a method derived from a validated, highly sensitive quantitative RT-PCR (qRT-PCR) shown to have a limit of detection of 20 parasites/ mL [23]. Total nucleic acid extraction was performed using the semi-automated NucliSENS easyMAG instrument (bioMerieux, Marcy l'Etoile, France), according to the manufacturer's instructions. Briefly, 50 µL DBS were hand cut with sterile scissors and added to 2 mL of NucliSENS lysis buffer, and the mixture was incubated for 30 min at 55 °C. The lysed sample was then transferred to the well of a plastic vessel with 70 μL of NucliSENS magnetic silica followed by automatic magnetic separation on the easyMAG to yield 40 μL of eluted nucleic acids.

Amplification and multiplex qRT-PCR were performed using the Applied Biosystems Quant Studio 5 Real-Time PCR System (Waltham, MA, USA). The thermal profile used for qRT-PCR was as follows: RT for 10 min at 48 °C, denaturation for 2 min at 95 °C, and then 45 cycles of 5 s at 95 °C for melting and 35 s 50 °C for annealing. Each reaction contained 7.5 µL of template total-nucleic-acid and a 17.5 µL reaction master mix containing 2 × SensiFAST Probe Lo-ROX Master Mix (Bioline/Meridian) with 0.5 µM of each primer and 0.25 µM of each probe. The pan-*Plasmodium-* and *P. falciparum*-specific probes and primers were previously described [23]. All qRT-PCR assays were run with appropriate controls including malaria positive, malaria negative and non-template controls. For quantification, copies/mL of whole blood were determined based on an absolute RNA calibrator curve and were also converted to estimated parasites/mL of whole blood by dividing by the per-parasite copy number conversion factor of 7.4 × 10^3^ 18S rRNA copies/parasite as previously reported [23]. If parasite densities were < 20 parasites/mL, qualitative results were reported: ‘low-positive’ for 10 to < 20 parasites/mL, and ‘not detected’ for any density lower than 10 p/mL or negative result.

### Study design, exposures and outcomes

A subset of samples and data from the 2015 MIS were analysed using a cross-sectional study design to evaluate whether subpatent *P. falciparum* infections were more common in households that included an RDT(+) individual compared to those with no RDT(+) individuals. There were 135 households randomly selected in which at least one member was RDT(+), and randomly matched them 1:4 to households with no RDT(+) individuals in the same district to balance the underlying prevalence in the sample. Households were eligible for selection into the analysis if at least two people were tested by RDT and at least one individual tested was RDT(−). Given this sample size and assuming that 15% of individuals in households without an RDT(+) individual had a subpatent infection, at alpha = 0.05, there was 80% power to detect a minimum prevalence odds ratio (POR) of 1.82 in the primary analysis. Not all samples with an RDT result in the MIS had a viable DBS for qRT-PCR analysis. As such, only matched sets in which each household had at least one qRT-PCR were included in the final analysis.

The main exposure of interest, living in a household with an RDT(+) individual, was generated using the RDT results from all individuals who were tested during the survey. A household was classified as having an RDT(+) individual if at least one member tested positive for falciparum malaria by RDT during the MIS, indicated by the presence of the HRP2 band. For the primary analysis, it was assumed that individuals who lived in the household but who were not present during the time of testing were RDT(−), and therefore would not influence the classification of the household. Individuals were defined as having a subpatent infection, the main outcome of interest, if they were RDT(−) for *P. falciparum,* but *P. falciparum* qRT-PCR positive, independent of the estimated parasite density.

## Statistical analysis

The association between living in a household with an RDT(+) individual and having a subpatent infection was evaluated using multivariate hierarchical logistic regression models [24] that incorporated inverse probability weights (IPW) to account for possible selection bias in household sampling. Models accounted for correlation at the household and community level as random effects and were adjusted for individual and household level covariates collected during the MIS that were considered a priori as potential confounders. Individual characteristics included a categorical variable for age, gender, recent travel to the mainland of Equatorial Guinea, and whether the individual reported sleeping under a bed net the previous night. Household factors included household size and the presence of open eaves. District was also included in the model as it was used to match households during selection.

**Fig. 2:**
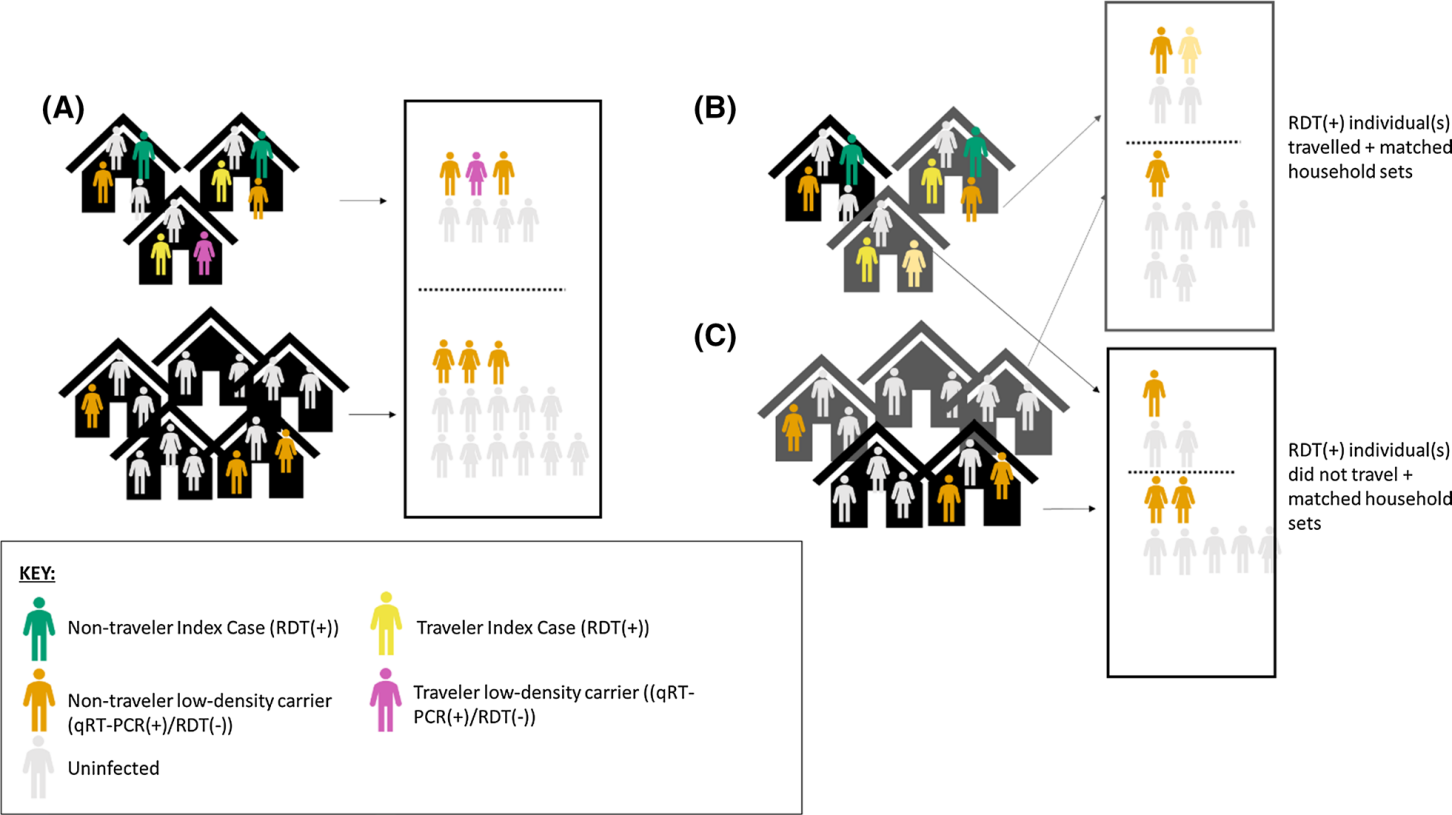
Schematic showing individuals included in the main (**A**) and stratified analyses (**B** and **C**). For the stratified analyses, individuals who live in a household with an RDT(+) individual who reports recent travel and their matched household sets (**B**) are analysed separately from individuals in households where the RDT(+) individual(s) did not report recent travel and their matched household sets (**C**)

Using data from all individuals in the full dataset, the probability that each individual was sampled by RDT was estimated using a generalized regression model accounting for categorical age, gender, off and on island travel history, and reported illness in the previous 14 days. Weights in the final model were the inverse of the selection probability for each included individual. All analyses were performed in R studio v.12.5033. Regression analyses were performed using the *lme4* package with the *optimx* or *bobyqa* optimizers. For each analysis, a minimally adjusted hierarchical model with no IPW, in which only district, the matching variable, was included, was evaluated. POR from this model as well as the inverse probability weight adjusted prevalence odds ratios (IPW aPOR) and associated 95% confidence intervals (CI) for each analysis are reported. To better understand the impact of suspected imported RDT(+) infections on household transmission, associations were further analysed based on the travel histories of the RDT(+) individual(s). Matched household sets were stratified according to the reported travel history of the RDT(+) individuals. Those in sets in which an RDT(+) individual had reported traveling off the island in the past eight weeks were analysed separately from the household sets in which the RDT(+) individual did not report recent travel (see Fig. 2). All reported travel in the past eight weeks was to the mainland of Equatorial Guinea. The same analytic approaches were repeated in both strata.

To evaluate the robustness of the assumptions regarding individuals who were not present in the home at the time of sample collection, several sensitivity analyses were conducted. In the primary analysis, it was assumed that individuals who were not present during the time of testing were RDT(−) and their presence would not change the exposure status of the household. To test this assumption, the RDT(+) probability for each individual missing an RDT test result in the sample was predicted using a hierarchical logistic regression prediction model. Using data from all individuals in the full MIS dataset with an RDT result, a predictive model that accounted for clustering at the household level and included a categorical variable for age, reported travel both on and off island in the past eight weeks, bed net use the previous night, presence of eaves in the household, household size and district was built. This model was then applied to the individuals in the sample dataset who had no RDT result to generate an RDT(+) predicted probability for each individual. Individuals with the highest predicted probabilities were assigned to be RDT(+) to achieve an overall RDT prevalence in the missing individuals of 5, 10 and 20%. These values were chosen to simulate a prevalence in the missing individuals that was similar to that seen in those tested (10%), similar to the maximum prevalence seen in the population (20%), or lower than what was seen in those tested (5%). The imputed RDT values were included with the observed RDT data to re-assign the exposure status of households as needed, and the full regression models were repeated with the imputed data sets.

Finally, to determine the impact of utilizing the highly sensitive qRT-PCR method compared to commonly used, less sensitive methods, an exploratory analysis was performed in which individuals with parasite densities < 1000 parasites/mL, the approximate limit of detection for newer ultra-sensitive RDTs [25], were reclassified as not infected.

## Results

**Fig. 3:**
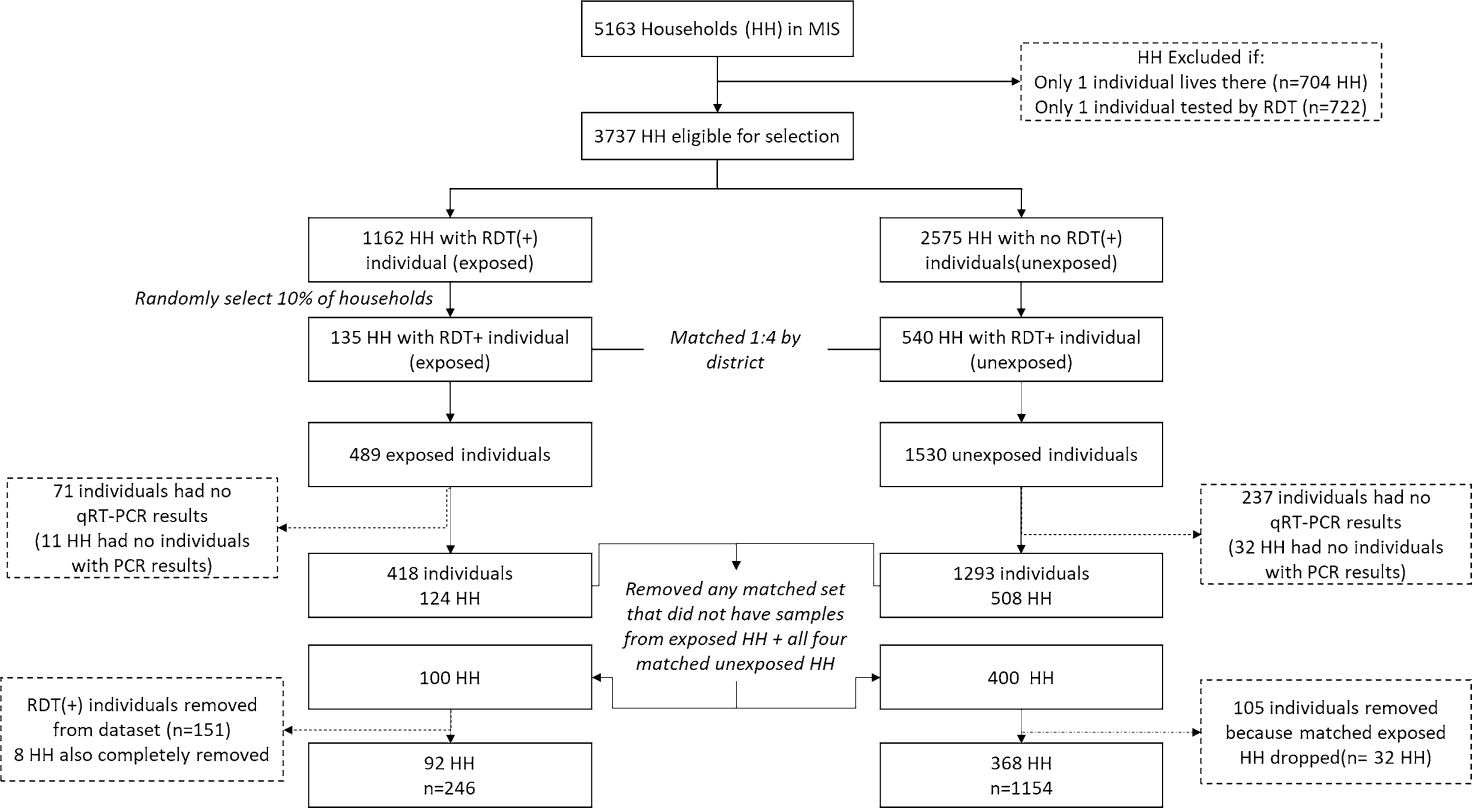
Flow chart showing how the final sample selection for analysis was determined (*HH* households, *MIS* malaria indicator survey, *RDT* rapid diagnostic test, *qRT-PCR* quantitative reverse transcriptase polymerase chain reaction)

Of the 5163 households sampled in the 2015 MIS, 3737 met the inclusion criteria for selection into the analysis (Fig. 3**).** Initially, 135 of the 1162 households with an RDT(+) individual and 540 matched households with no RDT(+) individuals were selected, which corresponded to 489 and 1530 individuals, respectively. After excluding individuals that had no qRT-PCR result (n = 308), and any matched household sets that did not have qRT-PCR results from all households in the set, the final dataset consisted of 246 individuals from 92 households with an RDT(+) infection, and 1,154 individuals from 368 households with no RDT(+) infections.

Select characteristics of households and individuals by household RDT status are summarized in Table 1. A higher proportion of households with an RDT(+) individual had open eaves and had a slightly larger household size compared to households without an RDT(+) individual. Individuals in households with an RDT(+) individual were slightly younger, with a higher proportion reporting travel in the past eight weeks, and a lower proportion reporting that they slept under a bed net the previous night compared to individuals in households with no RDT(+) individuals.

**Table 1:** Comparison of general characteristics of individuals who were RDT(−) during the MIS, and either lived in a household (HH) with an RDT(+) individual (94 HH) or did not (361 HH)

	RDT(+) in HH (n = 246)	No RDT(+) in HH (n = 1154)	All (n = 1400)
Individual characteristics			
Age (years)	17.3 (15.5); 0.1–76	19.1 (16.9); 0–88	18.8 (16.7); 0–88
Haemoglobin (mmHg)	12.0 (1.8); 5.7–16.9	12.3 (1.6); 5.5–18.9	12.2 (1.7); 5.5–18.9
Slept under net the previous night	122 (49.6%)	688 (59.6%)	810 (57.9%)
Was sick in past 14 days	12 (4.9%)	74 (6.4%)	86 (6.1%)
Travelled off-island in past 8 weeks	50 (20.3%)	114 (9.9%)	164 (11.7%)
Male	96 (39.0%)	495 (42.9%)	591 (42.2%)
Household characteristics			
Household size^2^	7.0 (2.9); 2–15	5.5 (2.2); 2–12	5.8 (2.4); 2–15
Proportion of household tested	0.7 (0.2); 0.1–1	0.7 (0.2); 0–1	0.7 (0.2); 0–1
Household prevalence by RDT	0.3 (0.2); 0–2	0.0 (0.0); 0–0	0.1 (0.2); 0–2
No screened windows	234 (95.1%)	1,101 (95.4%)	1,335 (95.4%)
Eaves are open	93 (37.8%)	266 (23.1%)	359 (25.6%)
Community characteristics			
Urban	193 (78.5%)	868 (75.2%)	1,061 (75.8%)
Malabo (Urban) District	167 (67.9%)	783 (67.9%)	950 (67.9%)
Malabo Periphery	26 (10.6%)	85 (7.4%)	111 (7.9%)
Baney District	15 (6.1%)	70 (6.1%)	85 (6.1%)
Luba District	26 (10.6%)	156 (13.5%)	182 (13.0%)
Riaba District	12 (4.9%)	60 (5.2%)	72 (5.1%)

^1^Statistics are N (%) for categorical variables and mean ± (SD); range for continuous variables

^2^Household size includes all individuals who normally sleep and eat in the house

^3^Acronyms: RDT = rapid diagnostic test; HH = household

Of the 145 samples from individuals who were RDT(+), 108 (74.5%) were also positive by qRT-PCR (see Additional File 1). Overall, there were 142 subpatent infections (10.0%) in the sample, with a mean density of 80,500 parasites/mL (range: 10–43.7*10^5^ p/mL). Of note, five such infections had high qRT-PCR densities, well above the limit of detection of an RDT. A greater proportion of individuals in households with an RDT(+) individual had subpatent infections than those in households with no RDT(+) infections (15.0 *vs* 9.1%). Among those with subpatent infections, there was no difference in the mean parasite densities between the two groups (50,800 p/mL *vs* 91,000 p/mL, p = 0.52). In the minimally adjusted, unweighted model, those living in households with an RDT(+) individual were 1.9 times as likely to have a subpatent infection than those who did not live with an RDT(+) individual (POR 1.93, 95% CI: 1.19, 3.14). In the fully adjusted model with IPW, the odds of having a subpatent infection were 2.6 times greater (aPOR 2.59; 95% CI: 1.31, 5.09) among those who lived in a household with an RDT(+) individual compared to individuals who lived in a household without RDT(+) individuals (Table 2).

**Table 2:** Inverse probability weighted adjusted prevalence odds ratios (POR) of a subpatent infection among individuals sampled in the 2015 Malaria Indicator Survey in Bioko Island

	All households (n = 1400)		Travel strata (n = 329)		No travel strata (n = 1071)	
	Minimally adjusted POR [95% CI]	IPW aPOR [95% CI]	Minimally adjusted POR [95% CI]	IPW aPOR [95% CI]	Minimally adjusted POR [95% CI]	IPW aPOR [95% CI]
Household characteristics						
Living in HH with RDT(+) individual	1.93 [1.19,3.14]	2.59 [1.31,5.09]	1.92 [0.84,4.39]	1.76 [0.54,5.67]	1.97 [1.1,3.54]	2.95 [1.17,7.41]
Having open eaves	–	1.74 [0.91,3.31]	–	2.33 [0.6,8.97]	–	1.61 [0.71,3.66]
Household size	–	0.98 [0.87,1.1]	–	0.96 [0.74,1.23]	–	1 [0.85,1.16]
Individual characteristics						
Slept under bed net previous night	–	1.13 [0.71,1.81]	–	0.92 [0.39,2.19]	–	1.2 [0.66,2.18]
Travelled off island in past 8 weeks	–	2.03 [1.14,3.6]	–	2.45 [0.92,6.57]	–	1.9 [0.85,4.22]
Male	–	1.52 [1.03,2.23]	–	1.31 [0.6,2.85]	–	1.72 [1.08,2.75]
Age under 5 years	–	Ref.	–		–	
Age 5–14 years	–	3.19 [1.46,6.94]	–	1.7 [0.46,6.24]	–	4.76 [1.68,13.46]
Age 15–44 years	–	9.16 [4.4,19.07]	–	2.95 [0.92,9.47]	–	17.53 [6.34,48.47]
Age 45 + years	–	4.19 [1.68,10.45]	–	4.16 [0.95,18.19]	–	4.61 [1.37,15.51]
Community characteristics						
Malabo (urban)	Ref.	Ref.	Ref.	Ref.	Ref.	Ref.
Baney	0.45 [0.16,1.28]	0.42 [0.11,1.54]	–	–	0.42 [0.14,1.25]	0.37 [0.08,1.68]
Luba	0.44 [0.21,0.92]	0.35 [0.14,0.88]	0.69 [0.09,5.47]	0.53 [0.04,6.26]	0.4 [0.18,0.89]	0.33 [0.11,1.01]
Riaba	1.22 [0.53,2.82]	0.95 [0.29,3.1]	0.87 [0.11,7]	0.61 [0.05,7.42]	1.27 [0.49,3.3]	1.09 [0.24,4.94]
Malabo (periphery)	0.86 [0.41,1.81]	0.79 [0.3,2.11]	1.52 [0.18,13.21]	1.44 [0.08,26.19]	0.78 [0.34,1.79]	0.69 [0.21,2.25]

Analysis by travel history to the mainland of Equatorial Guinea was conducted based on the travel status of individuals who were RDT(+). There were 145 individuals in 92 households who were RDT(+). Of these, 40 individuals (27.6%) from 27 households reported travel to the mainland of Equatorial Guinea in the past eight weeks. Individuals in households with RDT(+) travelers (n = 57) were analysed separately from those in households with RDT(+) non-travellers (n = 189), along with their matched household sets. Among the strata in which an RDT(+) individual had recently travelled, 15.8% of individuals in households with RDT(+) individuals had a subpatent infection compared to 8.8% in households without an RDT(+) individual. In the minimally adjusted, unweighted model, those living in households with an RDT(+) individual were 1.92-times as likely to have a subpatent infection than those who did not live with an RDT(+) individual; however, the result was not significant (95% CI: 0.84, 4.93). In the fully adjusted model with IPW, the odds of having a subpatent infection was 1.76-fold that of those living in homes with an RDT(+) individual compared to those in homes without RDT(+) individual, but the result was again not significant (95% CI: 0.54, 5.67) (Table 2).

Among the strata in which an RDT(+) individual had no reported recent travel, 14.8% of individuals in households with RDT(+) individuals had a subpatent infection compared to 9.2% in households without an RDT(+) individual (minimally adjusted POR 1.97, 95% CI: 1.1, 3.54). In the fully adjusted model with IPW, the odds of having a subpatent infection in those living in a household with an RDT(+) individual was 2.95-fold greater than in homes without RDT(+) individuals (95% CI: 1.17, 7.41) (Table 2). These results suggest possible modification of the relationship between subpatent and patent infections by the recent travel history of the RDT(+) individual in the household, as the association is stronger in households in which there is no history of travel.

**Fig. 4:**
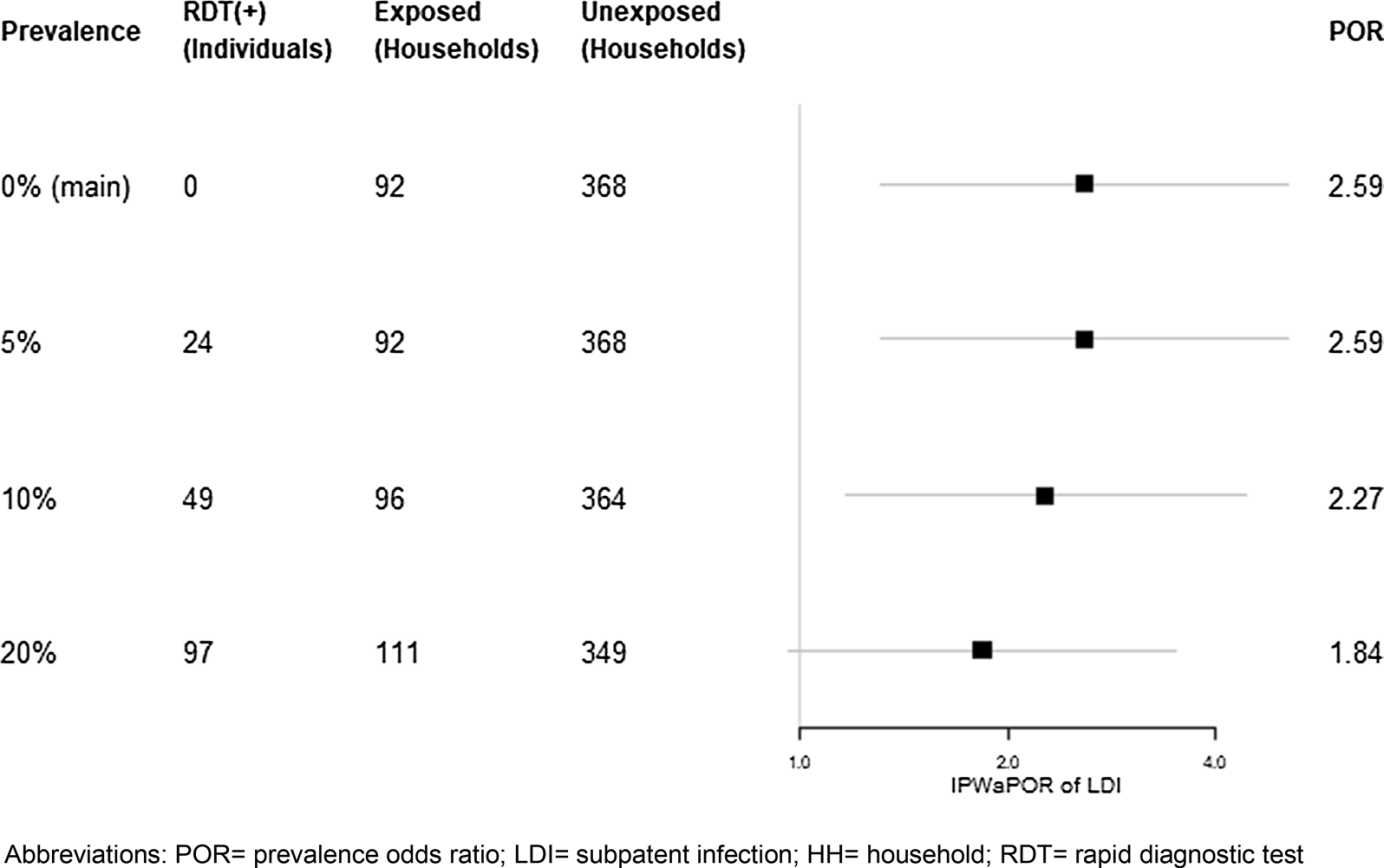
Inverse probability weight adjusted prevalence odds ratios (IPWaPOR) from sensitivity analyses which assumed various numbers of individuals missing from the main analysis were RDT(+), to achieve a range of plausible prevalences in the missing group.* POR* prevalence odds ratio, *LDI* subpatent infection, *HH* household, *RDT* rapid diagnostic test

The primary analysis assumed that individuals who were absent during the time of the survey were RDT(−). To test the robustness of this assumption, a series of sensitivity analyses were conducted using imputed RDT values for the individuals who were missing test results. Various scenarios of plausible ranges of RDT positivity in those who were missing were evaluated. There were 497 individuals from the households in the primary analysis who did not have an RDT result (22% of all individuals). Of these, 113 were from households with an RDT(+) individual and 384 were from households with no RDT(+) individuals. Compared to individuals who were tested, those who were not tested were older (mean age 29.0 years *vs* 17.7 years), had a higher proportion who were male (60.6 *vs* 41.9%), and reported more off-island travel (24.0 *vs* 12.0%). Figure 4 presents the modelling results based on imputed RDT(+) values in those with missing data at various levels of prevalence. As the presumed prevalence of infection in those missing RDT results increased, there was a slight decrease in the association between subpatent infection and living in a household with an RDT(+) individual. Only when 20% prevalence was assumed in those who were missing did the effect estimate fail to reach significance (POR 1.84, 95% CI: 0.96, 3.51).

Finally, to evaluate whether a similar association would be detected using other detection methods that are more sensitive than conventional RDT but less sensitive than the qRT-PCR method, a model was run to evaluate whether the association held when individuals with subpatent infections with densities below 1000 parasites/mL (approximate limit of detection of ultrasensitive RDTs) were classified as RDT(−). There were 35 individuals with infections < 1000 p/mL who were reclassified from subpatent infection to negative infection, 9 from households with RDT(+) individuals and 26 from households with no RDT(+) individuals. With this new classification, 11.4% of individuals who lived in households with an RDT(+) individual had a subpatent infection, compared to 6.8% of individuals who lived in a household without an RDT(+) individual. The adjusted IPW POR were similar to that of the main analysis, but the confidence interval was wider (aPOR 2.42, 95% CI: 1.02, 5.74).

## Discussion

In this analysis of the relationship between subpatent *P. falciparum* and RDT(+) *P. falciparum* infections from the 2015 MIS data on Bioko Island, living in a household with an RDT(+) household member was associated with increased odds of having a subpatent *P. falciparum* infection compared to living in a household with no RDT(+) household members. When stratifying by the travel history of the RDT(+) individual, the association was strongest among households in which the RDT(+) individual did *not* recently travel, while the association was attenuated and non-significant in the strata in which the RDT(+) individual travelled.

The results of the main analysis are similar to those observed in the highlands of Kenya, an area of low, but heterogeneous transmission [26]. In that area, where falciparum malaria prevalence by microscopy was estimated to be 6%, subpatent infections were 1.7 (95% CI: 1.6–1.8) times as likely to be in found in a household with a sentinel case, defined as an RDT(+) symptomatic adult or child or an RDT(+) asymptomatic child. In Zanzibar, where transmission is at pre-elimination levels, the association was even greater; the odds of having a subpatent infection were 7.4-times as great (95% CI: 2.8–19.9) for individuals living in a household with an RDT(+) individual compared to those living > 1000 m from the index case [27]. A recent meta-analysis of data from 17 different studies utilizing a mass test and treat strategy reported significant clustering of subpatent infections in households with RDT(+) individuals, and the degree of clustering increased linearly with decreasing prevalence [10]. These studies, along with data from the present analysis, suggest that household clustering is important to consider for malaria control strategies across a broad range of transmission settings.

Imported infections are a threat to malaria elimination efforts [13–17], especially if they are imported to areas conducive to onward transmission. When household sets were stratified based on the travel history of the RDT(+) individual, the association between subpatent infection and RDT(+) individuals was strongest in the strata in which there was no recent travel by the RDT(+) individuals. While an association was still suggested in households where importation of the infection was suspect, the association was attenuated and no longer significant. If it is assumed that reported travel in the past eight weeks is an indicator that the infection was imported, the attenuated relationship observed in the travel strata adds support to the hypothesis that imported cases may be returning to areas less receptive to propagation of infection, and therefore there may be little onward transmission of those infections [18]. Interestingly, the proportion of subpatent infections in households with and without RDT(+) cases was similar in the travel and non-travel strata, but after adjusting for relevant covariates, including travel status of the RDT(−) individuals, the association was stronger in the non-travel strata and attenuated in the travel strata. The most likely explanation for this is the high amount of travel in individuals with subpatent infections in households with RDT(+) travelers. Of nine individuals with subpatent infections in households with an RDT(+) traveller, seven (78%) also reported recent travel, compared to 21% of subpatent infections in households without an RDT(+) individual (n = 24). There was no difference in reported travel among individuals with subpatent infections in the non-travel strata. This may suggest that the clustering of subpatent infections in households with travellers is the result of multiple imported infections, while subpatent infections in households without recent travel are primarily due to local acquisition and transmission. A similar pattern was seen in a recent study in Zanzibar, an area with very low transmission. In that analysis, the odds of subpatent infection in household members of symptomatic RDT(+) cases who had recently travelled were 1.4 times that of clusters around locally acquired symptomatic RDT(+) infections; however, this association was largely driven by increased risk of subpatent infection of co-travellers of the index case [28]. In households where the index case was imported, the odds of subpatent infection was 2.5-times as great among co-travelling household members compared to non-travelling members. Given the study design and sample size, it was not possible to further delineate the patterns of transmission, and further studies designed to evaluate this are warranted.

Mass test and treat (MTAT) programmes in which RDT(+) infections are actively identified and treated in communities have shown to have minimal impact on patent malaria prevalence in both low [29] and high [30] transmission settings, which is thought to be due to the high prevalence of subpatent infections that are missed with this type of strategy. Additionally, these strategies rely on individuals being present at the time of testing, which pose logistical challenges, especially in urban areas, and may miss frequent travellers. A presumptive household treatment strategy in which all members of a household of an RDT(+) infection are provided treatment, independent of their RDT measured infection status, may better target subpatent infections and more effectively interrupt transmission [10]. In Bioko Island, this type of strategy may be most beneficial during localized outbreaks, in which a quick response is needed following an uptick in malaria cases [4]. Further, in areas of low-transmission where risk of malaria importation is high, strategies that aim to treat all household members of index patients who have recently travelled have been suggested [28]. This analysis suggests that a programme like this carried out in an area of moderate malaria transmission, such as Bioko Island, may have an impact on the overall parasite burden in the area, and would be beneficial independent of the recent travel status of the index case. In an area such as Bioko, officials might also consider providing treatment to all family members of travellers who are screened with an RDT on return and test positive. Even with measures like this, it should be noted that a number of subpatent infections in households without RDT detectable infections were also observed in this study, which may not be targeted with this type of approach.

In addition to living in a household with an RDT(+) individual, this analysis suggested that males and older individuals, especially those between 15 and 44 years of age, were most likely to have a subpatent infection, which is similar to what has been seen in other transmission areas [8]. It has been suggested that this group is most likely to have subpatent infections because of behaviours that increase likelihood of exposure, such as employment or greater time spent outdoors at night [31–33]. These factors may increase their cumulative exposure to malaria, which increases the likelihood they have built up a sufficient antibody response to control recent infections and keep parasite densities low [34]. Interestingly, a recent prospective analysis of *P. falciparum* infections in Uganda challenged this theory by showing that females cleared subpatent chronic infections twice as quickly as males [35]. Given that most data that compare subpatent and patent infections, including this study, come from cross-sectional studies, if there is a true difference in the rate of parasite clearance between males and females, it is possible that the increased risk reflects length-biased sampling instead of changes to the immune response. Whether the association is due to a true increase in risk or a sampling bias, the associations may still be beneficial in devising strategies that can target subpatent infections without utilizing ultra-sensitive diagnostic tools.

This study has several strengths, including the use of a highly sensitive qRT-PCR detection method, and the use of inverse probability weighting to account for selection bias that occurs during MIS sampling. However, these findings should be interpreted in the context of several limitations. While IPW was used to account for the missing data for subpatent infections, it was not possible to account for the possibility that individuals who were absent during testing were RDT(+), which may influence the exposure status of the household. In sensitivity analyses, the RDT prevalence in individuals missing from the survey would have to be > 20% before association between subpatent infections and RDT(+) cases disappeared. Given that the RDT prevalence in the sample was 8.5%, and the RDT prevalence estimated for the survey was 12.7% (95%CI: 12.0–13.4%), it is unlikely that the RDT prevalence in individuals who were not surveyed would approach 20%. While some RDT positivity in missing individuals attenuated the association, it was not substantial enough to alter the conclusions.

Another limitation is the utilization of travel history as a proxy for an imported infection. In the present study, travel history was based upon reported travel to the mainland of Equatorial Guinea in the past eight weeks. While this measure has been used to evaluate imported infections in other settings [36], there are several limitations that should be considered. First, given that a *P. falciparum* infection, on average, takes 12 days to be detected in blood in malaria-exposed populations [37], and the average time to naturally clear an infection is estimated between 87 and 200 + days [35], recent travellers could experience either higher or lower density infections, depending on date of their return and the date they acquired the infection in relation to the time of the survey. In an attempt to account for this possibility, recent travel of the RDT(−) individuals was controlled for, but it is still possible that some imported infections were missed. Secondly, given the large time span of travel, it is not possible to definitively determine if someone’s infection was acquired outside of the island or locally with the present study design. Frequency of travel and the amount of time spent in higher prevalence areas, as well as protective measures that were taken while travelling all might impact the probability that the RDT infection was imported. The time since return to the island and malaria risk behaviours exhibited at home might impact the risk of a locally acquired infection in a traveller since their return. Finally, in addition to travel to the mainland of the country, within-island travel is also common in Bioko, and increases the likelihood that individuals become infected outside of their communities. Without the use of parasite DNA sequencing or a prospective study design, some RDT infections may have been incorrectly classified in the stratified analyses. It is most likely that this misclassification would be non-differential and therefore correction of the misclassification would only strengthen the results. However, it is possible that there is differential misclassification, but without additional variables it is unclear the impact on the results.

Finally, the study defined a positive case based on the result of an RDT. However, qRT-PCR results showed that one-quarter of RDT(+) infections had no detectable parasitaemia, most likely the result of persistent HRP2 antigenaemia following clearance of an infection. While it is possible that inclusion of these individuals could impact the results, only one of these infections was in a household without another RDT(+) infection with detectable parasitaemia, and therefore exclusion of these individuals would not change the exposure status of the households in this analysis. There were also five individuals who were RDT(−), but had qRT-PCR defined parasitaemia above the limit of detection of RDTs, and would be expected to be RDT(+). A study in the mainland of Equatorial Guinea confirmed that *hrp2* deletion is present in the country [38] and may be the source of false negatives in the study population. While three of these infections were in individuals who did not live in a household with an RDT(+) individual, and therefore, the exposure status of the household may have been impacted if a different RDT was used, the small number of false positives would not be expected to impact our results. Given that RDTs are most commonly used for detection of infection in the study setting, our results represent the associations that are seen with these widely deployed diagnostic tools.

## Conclusions

Subpatent infections of falciparum malaria infections are more prevalent in households with an RDT(+) individuals in Bioko Island, Equatorial Guinea. The association is present both when local and imported RDT infections are suspected, albeit stronger in households where local acquisition is suspected. These findings support the need for possible malaria control strategies that treat household members of RDT(+) individuals to target subpatent infections and decrease the *P. falciparum* infectious reservoir.

## Supplementary Information

**Additional file 1. **Concordance between rapid diagnostic test (RDT) *Plasmodium falciparum *(Pf) positivity and quantitative reverse transcriptase polymerase chain reaction (qRT-PCR) *P. falciparum* positivity, for select samples from the Malaria Indicator Survey (MIS) from Bioko Island, Equatorial Guinea, 2015 (n=1650). Results include individuals from all households tested, before exclusions were made to reach the final analytic sample.

## Data Availability

The datasets used and/or analysed during the current study are available from the corresponding author on reasonable request.

## Abbreviations

ACT: Artemisinin-combination therapy
aPOR: Adjusted prevalence odds ratio
BIMCP: Bioko Island Malaria Control Programme
CI: Confidence interval
DBS: Dried blood spot
IPTp: Intermittent preventive treatment in pregnancy
IPW: Inverse probability weight
IRS: Indoor residual spraying
LLIN: Long-lasting insecticidal nets
MIS: Malaria Indicator Survey
MoHSW: Ministry of Health and Social Welfare
MTAT: Mass test and treat
NMCP: National Malaria Control Programme
p: Parasites
POR: Prevalence odds ratio
qRT-PCR: Quantitative reverse transcriptase polymerase chain reaction
RDT: Rapid diagnostic test
