# Supplementary material for: Clustering of subpatent infections in households with asymptomatic rapid diagnostic test-positive cases in Bioko Island, Equatorial Guinea independent of travel to regions of higher malaria endemicity: a cross-sectional study
Source: Malar J. 2021 Jul 12;20:313. doi: 10.1186/s12936-021-03844-6 (PMC8274032; doi:10.1186/s12936-021-03844-6)
Supplement: Supplementary file 1 — Additional file 1. Concordance between rapid diagnostic test (RDT) Plasmodium falciparum (Pf) positivity and quantitative reverse transcriptase polymerase chain reaction (qRT-PCR) P. falciparum positivity, for select samples from the Malaria Indicator Survey (MIS) from Bioko Island, Equatorial Guinea, 2015 (n=1650). Results include individuals from all households tested, before exclusions were made to reach the final analytic sample. [file 12936_2021_3844_MOESM1_ESM.docx]

|  |  | **RDT** | |
| --- | --- | --- | --- |
|  |  | **Pf(+)** | **Pf(-)** |
| **qRT-PCR** | **Pf +** | 108 | 160 |
|  | **Pf (-)** | 37 | 1345 |
|  | **TOTAL** | **145** | **1505** |

**Additional file 1.** Concordance between rapid diagnostic test (RDT) *Plasmodium falciparum (Pf)* positivity and quantitative reverse transcriptase polymerase chain reaction (qRT-PCR) *Pf* positivity, for select samples from the Malaria Indicator Survey (MIS) from Bioko Island, Equatorial Guinea, 2015 (n=1650). Results include individuals from all households tested, before exclusions were made to reach the final analytic sample.
